# Tracking dynamics of plant biomass composting by changes in substrate structure, microbial community, and enzyme activity

**DOI:** 10.1186/1754-6834-5-20

**Published:** 2012-04-10

**Authors:** Hui Wei, Melvin P Tucker, John O Baker, Michelle Harris, Yonghua Luo, Qi Xu, Michael E Himmel, Shi-You Ding

**Affiliations:** 1 Biosciences Center, National Renewable Energy Laboratory, Golden, CO 80401, USA; 2 National Bioenergy Center, National Renewable Energy Laboratory, Golden, CO 80401, USA

**Keywords:** Compost, Plant biomass, Yellow poplar, Microbial community, Microbial rDNA abundance, Gene expression, Enzymatic activity, Cellulase, Hemicellulose, Ligninase, Consolidated bioprocessing (CBP), Solid-state fermentation, Biofuels

## Abstract

**Background:**

Understanding the dynamics of the microbial communities that, along with their secreted enzymes, are involved in the natural process of biomass composting may hold the key to breaking the major bottleneck in biomass-to-biofuels conversion technology, which is the still-costly deconstruction of polymeric biomass carbohydrates to fermentable sugars.

However, the complexity of both the structure of plant biomass and its counterpart microbial degradation communities makes it difficult to investigate the composting process.

**Results:**

In this study, a composter was set up with a mix of yellow poplar (*Liriodendron tulipifera*) wood-chips and mown lawn grass clippings (85:15 in dry-weight) and used as a model system. The microbial rDNA abundance data obtained from analyzing weekly-withdrawn composted samples suggested population-shifts from bacteria-dominated to fungus-dominated communities. Further analyses by an array of optical microscopic, transcriptional and enzyme-activity techniques yielded correlated results, suggesting that such population shifts occurred along with early removal of hemicellulose followed by attack on the consequently uncovered cellulose as the composting progressed.

**Conclusion:**

The observed shifts in dominance by representative microbial groups, along with the observed different patterns in the gene expression and enzymatic activities between cellulases, hemicellulases, and ligninases during the composting process, provide new perspectives for biomass-derived biotechnology such as consolidated bioprocessing (CBP) and solid-state fermentation for the production of cellulolytic enzymes and biofuels.

## Background

The intertwining matrix of biopolymers (celluloses, hemicelluloses, and lignins, as prominent examples) of which plant cell walls are composed poses a major obstacle to the deconstruction of these walls to simple sugars and chemicals that can serve as raw materials for the fermentative production of alternative liquid fuels and other bioproducts. This major bottleneck in biomass conversion technology can be mitigated by 1) reducing plant biomass recalcitrance through genetic engineering of energy crops, thereby 2) minimizing the requirement for thermo-chemical feedstock pretreatment, 3) improving performance of the enzymes used for saccharification, and 4) introducing the one step conversion concept, or consolidated bioprocessing (CBP), in which enzyme production, enzymatic hydrolysis, and fermentation are combined for microbial production of biofuels using biomass as substrates [1].

Traditionally, composting is defined as a process that heaps together organic materials (notably food waste, manure, plant leaves and stems, grass trimmings, crop residues, paper, and wood, etc.), and allows them to decay enough to be ready either for use as soil enhancers or for disposal. This composting process mainly depends on microorganisms (including archaea, bacteria and fungi) to break down the organic materials. In the past decades, composting has evolved from a means for the management of agricultural and residential waste [2,3], or for their conversion of into value-added products such as fertilizers [4,5] to a tool used to mine for novel microorganisms and enzymes to be applied to the conversion of plant biomass to biofuels [6,7].

The advantages provided by the study of composting systems (vs. study of other ecosystems such as soils and wood/leaf litters) include: 1) allowing more control over the exo-environmental conditions, such as external temperature, moisture, etc., and 2) generation of a more diverse microbial community because of the steeply decreasing oxygen gradient from outermost to innermost layers of a compost pile [8]. These advantages make composting studies a rich area to be mined for activities useful in both biological pretreatment of biomass feedstocks and in the ultimate saccharification step.

It is a generally accepted idea that environments and cell-cell interactions shape the species composition in communities [9-11]. In addition, numerous published reports have revealed that the diversity of microbial communities and their secreted enzymes that are involved in degradation is correlated with biomass type (different tissue types or plant species) [see review [8]], which suggests that composting of a recalcitrant lignocellulose-based biomaterial is more likely to lead to a microbial community with higher capacity in degrading plant cell walls, when compared to composting of other more readily degraded materials such as kitchen food wastes. From this perspective, the fact that the biomass of woody energy crops (such as yellow poplar) has significantly higher recalcitrance indices (with values of 0.56-0.87) than those of herbaceous energy crops (0.25-0.45; [8]), suggests that knowledge derived from yellow poplar-composting can be directly and effectively applied to the conversion of woody plant biomass to simple sugars.

Most previous studies of composting have mainly focused on characterizing the microbial composition of the composting communities [3,12-14], with only a few focused on the morphological changes in the surface structure of plant biomass substrates [15], and even fewer on the characterization of the cellulolytic enzymes and their encoding genes, let alone the correlations between the above multiple aspects of composting. Recent metagenomic studies of soils and biomass composts [6,9,16] have yielded significant insights into uncultivated microbial communities; these studies, however, have investigated only single or a few sampling time points, probably due to the considerable cost and labor-intensity of analyzing the metagenomic data.

The major objectives of the current study were to fill the above voids by conducting, for the first time, a timeline characterization of the yellow poplar wood-chip-based biomass decay community. We hypothesize that, in the biomass composting ecosystem, the microbial community involved in the deconstruction process of biomass is dynamically correlated with the status of biomass substrates, as well as with environmental and timing factors. To test this hypothesis, comprehensive, multi-directional approaches are employed herein to draw time-course correlations between the microbial composition, functional gene expression, cellulolytic enzyme activity, and plant cell wall structural changes.

## Results

**Figure 1:**
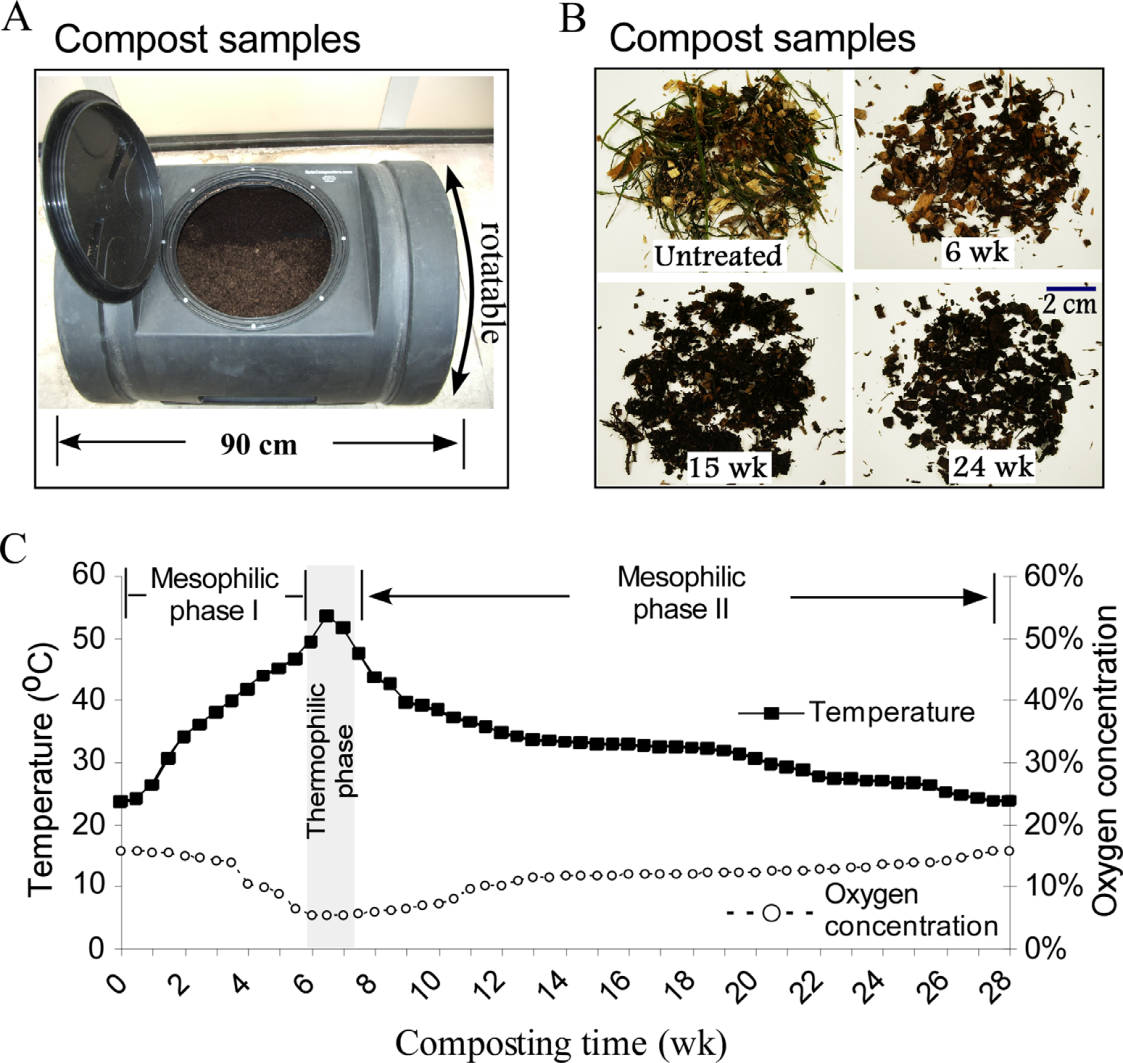
**Compost setup and sampling**. (**A**) The apparatus for composting of yellow poplar wood chips. (**B**) Representative samples collected showing morphological changes of composted poplar chips, i.e., size reduction, color darkening, and material softening. (**C**) Temperature and oxygen concentration measured, as described in Materials and Methods, during composting process. wk: week.

As described in the Materials and Methods section, the composting bin (Figure 1) was set up indoors, and temperature and oxygen levels in the composting mass were regularly monitored. Figure 1C shows a typical compost pattern [3] with three stages. Initially a mesophilic phase I (20°-50°C) of 6 weeks was observed, followed by a brief thermophilic phase (one week above 50°C). Thereafter, the temperature returned to the mesophilic range until the composting finished.

Conventional composts usually contain highly nitrogenous materials, such as sewage and manures, in relatively large proportions, and thus tend to produce more heat and result in higher temperatures, up to 80°C [3], in the thermophilic phase. In contrast, our biomass compost contained less nitrogen and generated less heat, resulting in lower composting temperature.

The ambient oxygen concentration at the center of composter basically had a negative correlation with the compost temperature (Figure 1C). When the temperature was at 50°-53°C during the thermophilic phase, the ambient oxygen concentration reached levels as low as 4%.

### Microscopic imaging reveals successive stages of deconstruction of composted biomass

**Figure 2:**
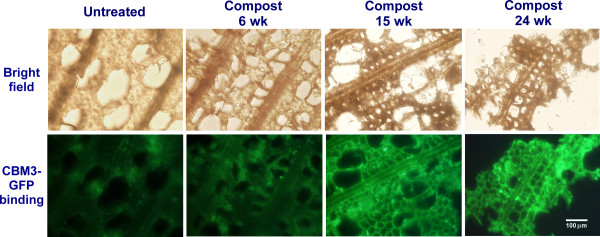
**Cross-section micrographs of yellow poplar wood chips**. (Top panel) Bright field microscopy shows composting effects on the cell wall structure over 24 weeks. (Bottom panel) fluorescence microcopy of the same field labeled by the *Ct*CBM3-GFP probe that binds to cellulose specifically. Increasing fluorescence intensity indicates higher cellulose accessibility to the probe. *Ct*CBM3-GFP: family 3a carbohydrate-binding module tagged by green-fluorescent-protein. wk: week.

Biomass was imaged at successive stages of decay using optical microscopy. Apparent decay of the plant cell wall structure was observed after 6 weeks of composting (Figure 2). In the bright field (Figure 2, upper panel), the overall structure of the plant cell walls has begun to collapse at 15 weeks, and substantial biomass loss was observed at 24 weeks. In addition, a green-fluorescence-protein tagged carbohydrate-binding module (CBM) was used as probe to localize cellulose, and labeled samples were then imaged with fluorescence microscopy. The family 3a CBM used in this study, termed *Ct*CBM3, was originally isolated from *Clostridium thermocellum *scaffoldin protein, and has strong binding affinity to crystalline celluloses [17-20]. Figure 2 (lower panel) shows that the *Ct*CBM3-GFP (green fluorescent protein) binding to yellow poplar cross-sections increased after 15 to 24 weeks of composting, suggesting that more hemicellulosic material was degraded in the early stages of composting, leading to progressively greater cellulose exposure and increased access for the *Ct*CBM3-GFP. Such observation is consistent with previous reports that removal of xylan enhances cellulose accessibility and digestibility [21].

### Compositional analysis for the composted materials

To assess the degradation effect of composting on the feedstocks, the compost samples collected at 1 and 27 weeks were used to measure the remaining amounts of cellulose, hemicellulose and lignin, along with other compositions, using the chemical analysis procedures described in Materials and Methods.

The results are shown in Table 1. Taking the data for week 1 samples as the "initial" numbers, we found that the cellulose content in compost samples changed from 39.2% to 19.5%, i.e. decreased by 50%. The contents of xylan and mannan, two major hemicellulose components, in compost samples changed from 13.9% to 7.1%, and from 2.3% to 1.4%, respectively. In other words, xylan and mannan decreased by 49% and 40%, respectively. Taken together, these data indicate that the rates of the decrease in amounts of cellulose, xylan and acetyl groups (which mainly link with or exist in xylan) are very similar, between 47% and 50%. This degradation effect is significant, and is comparable with the literature reported recalcitrance index (RI) value for hardwood biomass yellow poplar, which is 0.56 (means 44% of biomass is degradable; see the Discussion section).

**Table 1:** Compositional data for the composted materials

Samples	% Structural Inorganics	% Non-structural inorganics	% Structural Protein	% Sucrose	%Water Extrac table Others	% Ethanol Extractives	% Lignin	% Glucan (cellulose)	Hemicellulose					% Acetyl groups	% Total
											
									% Xylan	% Mannan	% Galactan	% Arabinan	% Subtotal hemicellulose		
wk 1	**1.7**	**1.1**	**0.7**	**3.3**	*1.2*	**1.6**	26.3	*39.2*	*13.9*	*2.3*	0.6	0.5	*17.3*	*3.4*	95.9

wk 27	**5.3**	**2.4**	**7.8**	**7.3**	*0.3*	**3.4**	35.8	*19.5*	*7.1*	*1.4*	0.9	0.6	*10.0*	*1.8*	93.7

Changes at wk 27 compared with wk 1								-50%	-4 9%	-40%				-42%	-47%

The data are averages of measurement of two replicate samples.

The **bold **numbers indicate the measurements that increase, while the *italic *numbers indicate the measurements that decrease during the composting process. wk, week

Contrary to the trends seen for hemicellulose and cellulose contents, an increase in the content of lignin was observed (Table 1). This is likely a result of the absolute amount of lignin staying the same while the absolute amounts of other components decrease, rather than of the generation of lignin in composting. The percent by weight of structural protein increased as well, the much larger proportional change (more than 10-fold) in this case likely reflecting actual increases in absolute amounts of composting organisms and enzymes.

Future study of chemical compositional analysis at more sampling time points will be helpful to provide deeper insights into the composting process.

### rDNA shifts reflect environmental and microbial-population shifts

**Figure 3:**
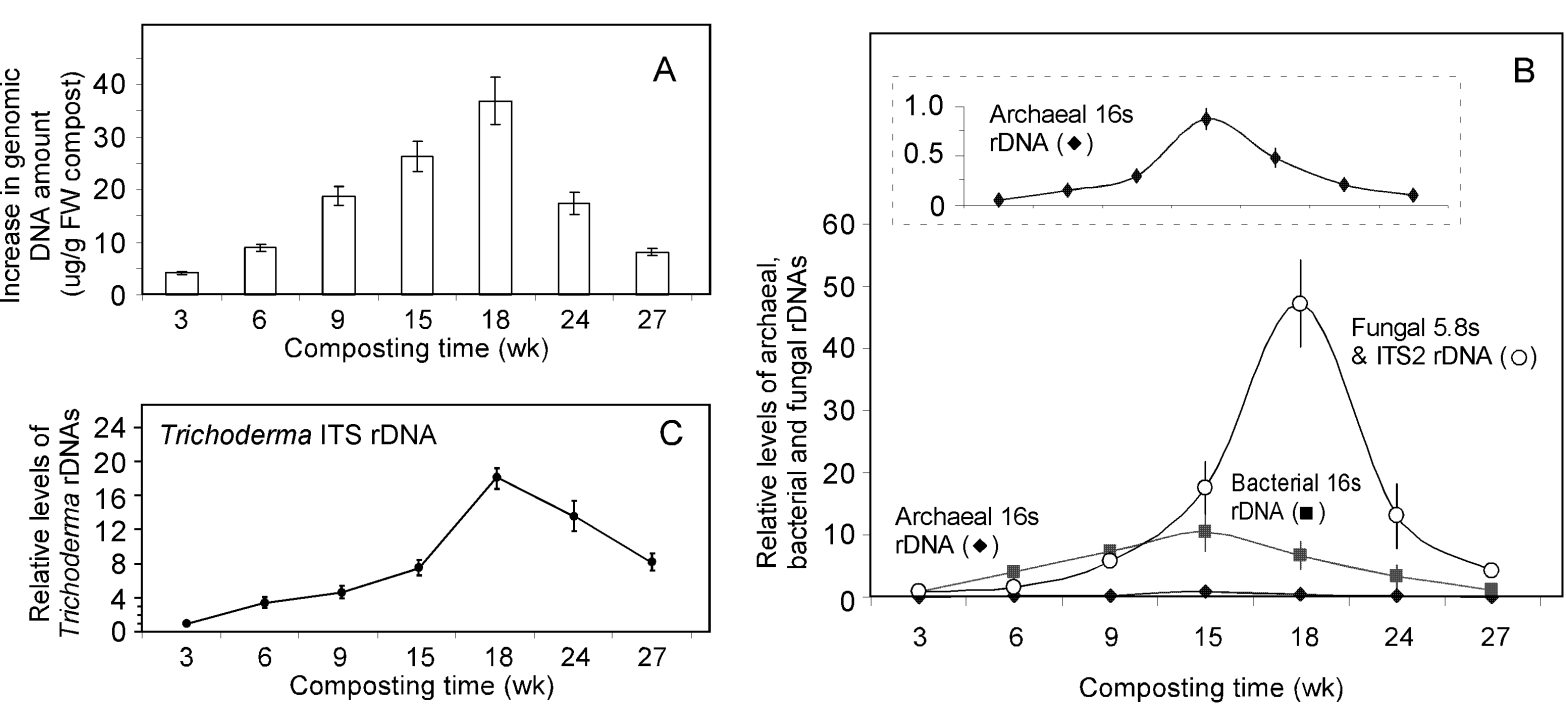
**Relative abundance of total genomic DNAs extracted from composted yellow poplar chips, and microbial rDNAs by PCR using primers in Table 2**. Samples were collected in composting time at 3, 6, 9, 15, 18, 24, and 27 weeks. (**A**) Amount of total genomic DNAs above the baseline genomic DNAs in compost at 1 week which was 32.7 ± 2.6 μg/g FW compost (FW: fresh weight). (**B**) Relative level of bacterial, archaeal and fungal rDNAs. 16 s rDNA was used in bacteria and archaea, and 5.8 s and ITS2 rDNA were used in fungi. The bacterial rDNA abundance at 3 weeks was set as 1- fold; archaeal and fungal rDNA relative level was adjusted to bacterial rDNA abundance at 3 weeks. The insert in (**B**) shows the archaeal rDNA profiling with a fine scale for the relative levels of archaeal rDNA. Note the bacteria-dominant stage at 9 weeks, whereas the fungi-dominant and overall peak stage occurs at 18 weeks. (**C**) Relative levels of *Trichoderma *spp. ITS rDNA. Error bars indicate the standard errors of the mean (S.E. ± mean) for the three replicates.

Samples from 3, 6, 9, 15, 18, 24, and 27 weeks of composting were collected for total genomic DNA extraction. Figure 3A shows that the amounts of total genomic DNA increase steadily along the time course of composting with a peak at 18 weeks, followed by a decline in 24-27 weeks. This result appears to be correlated with the recorded drop in temperature during the late stages of composting (Figure 1C).

Like many other environmental samples, extracts from composted biomass materials may contain high concentrations of organic matter. For example, humic acids commonly persist in isolated genomic DNA, and can be inhibitory to PCR and thereby compromise the quantitation of rDNA abundance. To address this issue, a serial dilution of isolated genomic DNA was tested to optimize the template concentration and to eliminate the effect of inhibitors. Using primers listed in Table 2, we found that genomic DNA concentrations between 0.08 and 2.5 ng per well (20 μl reaction volume) resulted in a linear relationship between Ct (cycle threshold, which is the main output of real-time PCR data, defined as the number of cycles required for the fluorescent signal to cross the threshold, i.e. exceed the background level) and the log of DNA concentration. The PCR amplification efficiency values, calculated as 10^(-1/*slope*) ^(in which the slope refers to the above-constructed Ct vs. the log of DNA concentration standard curve), were calculated to be 1.72, 1.80, and 1.81 for the archaeal, bacterial, and fungal rDNA primers, respectively. Note that a PCR amplification efficiency value of 2 means 100% success in PCR amplification. The obtained amplification efficiency values are comparable to those in other reports using the same or similar universal primers [22-24]. These values were used to calibrate the PCR-based measurement of rDNA abundance in this study.

**Table 2:** Domain-level primers used to amplify short-subunit rDNA genes from DNA extracts of biomass compost

	Genes	Primer sequences & reference	Amplicon size(bp)
Archaea	16 s rDNA	F: TTCCGGTTGATCCYGCCRG R: YCCGGCGTTGAMTCCAATT Ref: [22]	937

Bacteria	16 s rDNA	F: TCCTACGGGAGGCAGCAGT R: GGACTACCAGGGTATCTAATCCTGTT Ref: [25]	466

Fungi	5.8 s and ITS2 rDNA	F: GCATCGATGAAGAACGCAGC R: TCCTCCGCTTATTGATATGC Ref: [22,26]	400

To assess the diversity of each group of microbes at each stage of the composting, real-time PCRs were conducted using 2.5 ng genomic DNA per reaction and universal primers for 16 s rDNA (bacteria and archaea) and 5.8 s and ITS2 rDNA (fungi) (Table 2). The archaeal, bacterial, and fungal rDNA relative abundance was first calculated with the delta-delta Ct method, using the bacterial rDNA level at 3 weeks as the common calibrator, and then normalized to the yield of total genomic DNA in each sample. The results are shown in Figure 3B. Interestingly, archaea and bacteria have similar patterns in rDNA abundance; both had a gradual increase with a peak at 15 weeks, followed by a decline until the end of composting. In contrast, fungi had a more distinctive pattern for rDNA abundance, which peaked at 18 weeks in a more abrupt rising and falling manner.

The observed higher proportion of fungi in the later stage of composting (Figure 3B) suggests that while bacteria may be more active when hemicelluloses are the easily accessible carbohydrates, fungi are more active when celluloses and lignins become exposed and accessible. The composting stages at week 9 and 18 therefore represent bacterial and fungal dominant phases, respectively (Figure 3B), and are candidate time points for sampling RNA for future metatranscriptomic analysis.

In addition, we also determined the relative abundance of *Trichoderma *spp. ITS rDNA during the time course of yellow poplar composting (Figure 3C), as a measure of the presence and abundance of microorganisms of genus *Trichoderma*. This paves the way for the profiling of functional gene expression for representative species in this genus, as described in later section.

In summary, the domain-level screening provided a strong timeline characterization for the composting process. The data from relative rDNA abundance for the microbial groups pointed to population shifts in the microbial composition during the composting process.

It is noteworthy that, in contrast to the dynamic changes in relative abundance of bacteria and fungi, archaea remain relatively stable in the amount of rDNA (1-2%) throughout the course of the composting. Archaeal mass found in present yellow poplar compost is similar to that reported in other ecosystems, such as agricultural and field soils [27-29], suggesting as-yet-unknown roles for archaea in biomass decay systems.

### Functional gene expression profiling

To deeply understand the dynamics of biomass composting, it is important to conduct functional expression profiling related specifically to the biomass-degrading process, namely of the known genes encoding cellulases, hemicellulases, and lignin-modification enzymes. However, such functional studies are challenging because of the vast variety in the types of cell-wall-degrading enzymes and the lack of experimentally-validated function annotations of related genes in public databases. As described above, the population dominance shifted from bacteria to fungi in later composting stage. We therefore focused on fungal genes. To this end, several model cellulolytic fungi for which genome sequences are available were selected for sequence alignment and primer design for RT-PCR of functional genes. Table 3 shows the list of primer sequences used for genes encoding cellulases, hemicellulases and β-glucosidases in the model aerobic fungus, *Trichoderma *spp., which is the dominant genus found in various biomass decay ecosystems, as well as being a common producer for most of the cellulase and hemicellulase enzymes used in industry), and for genes apparently encoding ligninase enzymes in the white-rot fungus *Phanerochaete chrysosporium *[30].

**Table 3:** Subgenus- and species-level primers used to determine the transcriptional levels of fungal cellulolytic genes in biomass compost

Enzyme type/core species	Target gene/enzyme	Genbank accession No. or reference	**Primer sequences ** [I]	Amplicon size (bp)
*Trichoderma *spp.	ITS	-	F: TACCAAHCTGTTGCCTCGGCGG	~200
				
			R: GATGAAGAAGGCAGCGAAATGCGATA	

Cellulase/*Trichoderma *spp.	*cbh*1/Cel7 A	[II]	F: GATGATTACTACGCCAACATGCTG	77
				
			R: ACGGCACCGGGTGTGG	
	
	*egl*1/Cel7 B	[III]	F: CTGCAACGAGATGGATATCCTG	250
				
			R: GTGATGATGGTGAAGGTCTTGGAG	
	
	*bgl*1/Cel3 A	[IV]	F: ATCAAGGTAGCTCAACATCGGG	124
				
			R: ACCTTATCTTGGAGATTGAGCTTTGCC	

Hemicellulase/*Trichoderma *spp.	*xyn*1 and *xyn*2	[V]	F: CCGAGAAGTTGATGACCTTGTTC	87
				
			R: GGTCCAACTCGGGCAACTTT	

Ligninase/*Phanerochaete chrysosporium*	LipA/B	M37701	F: ATCTCTGCCCACCCTGTCCT	256
				
			R: CTGAGCCAGCGAATGAGAGTC	
	
	LipD	M18743	F: CCCGGTCCTCGATGATATCC	201
				
			R: ATGTTCGGGTGGTACGTGGT	
	
	LipH	M24082	F: CGTCCACGGATATCGCTCTCT	102
				
			R: GCGAGGGAGACGCAAATTC	
	
	LipJ	AF140062	F: GCCGAGGCACATGAGTCTCTC	252
				
			R: TGTTCGGGTGGAAATTGGTC	

Note: the group primers targeting specific functional protein-encoding genes:

[I]. *Orpinomyces *spp. endo-1,4-glucanase gene: *O*. sp. PC-2, U97153; *O. joyonii*, AF015248.

[II]. *Trichoderma *spp. cellobiohydrolase I gene (*cbh*1): *T. koningii*, X69976; *T. reesei*, DD393553-DD393571; *T*. sp. FJ026620; *T. viride*, AY368686.

[III]. *Trichoderma *spp. endo-β-1,4-glucanase I gene (*egl*1): *T. longibrachiatum*, X60652; *T. reesei*, AY928809; *T. viride*, AY343986

[IV]. *Trichoderma *spp. β-glucosidase 1 gene (*bgl*1): *T. reesei*, U09580; *T. sp*. SSL, FJ040193; *T. viride*, AY368687

[V]. *Trichoderma *spp. *xyn*1 and *xyn*2: *T.reesei*, X69573 (*xyn*1), U24191 (*xyn*2); *T. harzianum*, EU821597 (*xyn*2); *T. pseudokoningii*, EU360941 (*xyn*2).

With the exception of those for *Trichoderma *sp. ITS rRNA [31], and *cbh*1/Cel7A and *bgl*1/Cel3A [adapted from literature [32]], and *xyn*1 and *xyn*2 of *Trichoderma *spp. [adapted from literature [33]], the primers employed were designed in this study.

Using the approaches described in the Materials and Methods section, expression levels of functional genes were calculated with the delta-delta Ct method, using *Trichoderma *spp. ITS (listed in Table 3) or fungal 5.8 s and ITS2 rDNA/rRNA (listed in Table 2) as an internal control for *Trichoderma spp*. and *Phanerochaete chrysosporium *genes, respectively. Each individual gene's mRNA level at 3 weeks was set as the reference value to calculate the subsequent fold changes.

### (i) Transcription-level profiling of fungal hemicellulases and cellulases reflects coordination of gene expression in targeting progressively degrading biomass substrates

*Trichoderma *is a genus of fungi that exists, and often predominates, in broad types of soils and diverse environments including composts. Most species of this genus, including the industrial cellulase producer *T. reesei*, are saprophytes that can degrade bio-polymeric substrates such as lignocelluloses. This prompted us to use this genus as a model group to investigate the transcriptional dynamics of hemicellulase- and cellulase-encoding genes during the composting process.

**Figure 4:**
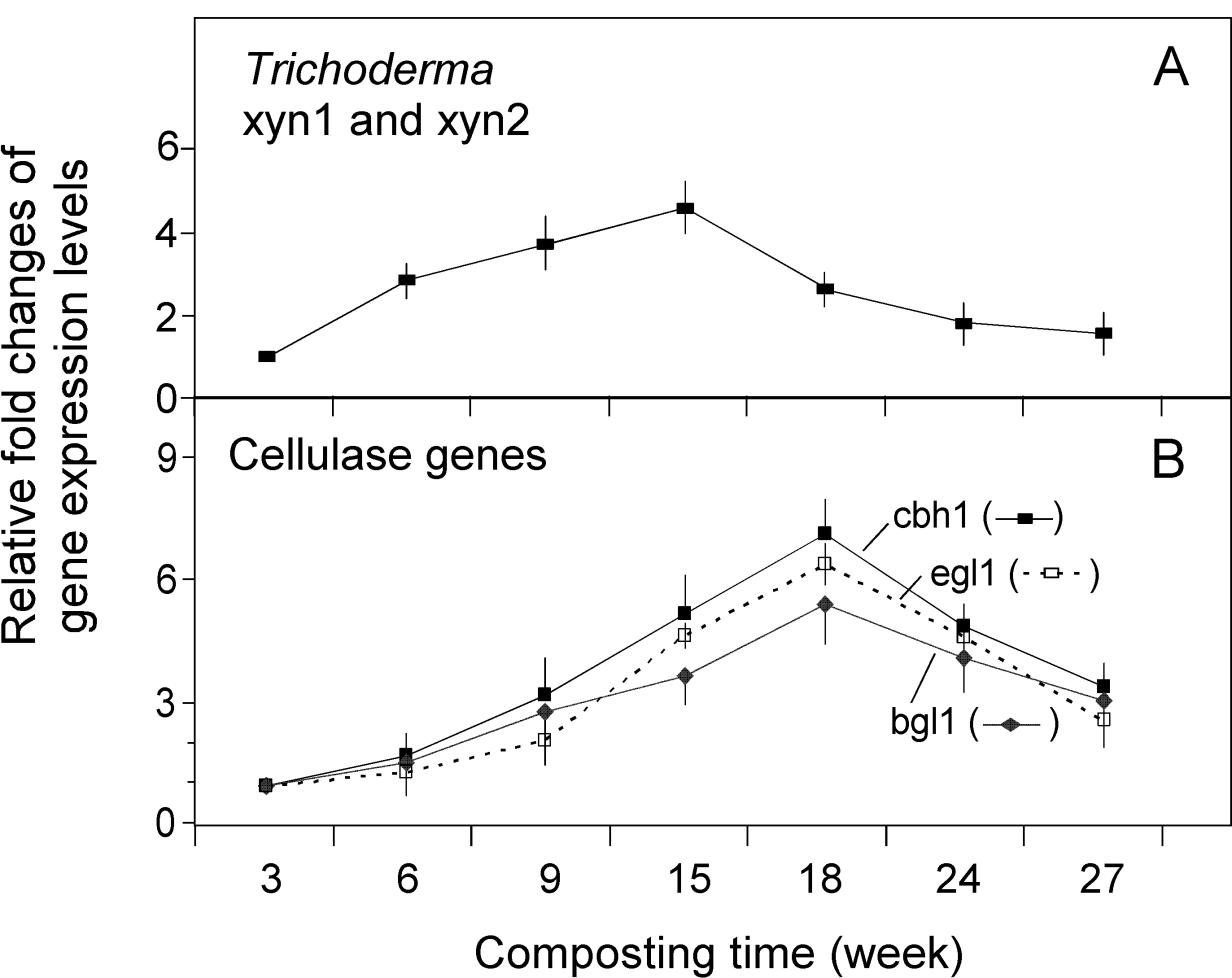
**Transcriptional level of representative cellulolytic functional genes in *Trichoderma *sp. by real-time RT-PCR during composting of yellow poplar chips**. (**A**) Xylanases 1 and 2 (*xyn*1 and *xyn*2). (**B**) Cellobiohydrolase I (*cbh*1), endoglucanase I (*egl*1) and β-glucosidase 1 (*bgl*1) were used in a set of representative species of *Trichoderma *genus. The gene expression level at each sampling time point of composting was first normalized with the *Trichoderma *sp. ITS rRNA, and then compared to their respective expression levels at 3 weeks (each of which was set as 1 fold). The primers for these genes were described in Table 3. Note that identical scales in the X axis of panels A-B allows a direct visual comparison of the magnitude of changes in gene expression levels.

To assess the relative expression levels of hemicellulases in composted samples, primers targeting both of the two major xylanases in *Trichoderma *(*xyn*1 and *xyn*2; Table 3) were used in real-time RT-PCR analysis. The results demonstrated that the expression levels for these xylanases steadily increased between 6 to 15 weeks of composting (Figure 4A), and then declined markedly after that.

Meanwhile, to assess the expression of cellulase genes of genus *Trichoderma *during the composting, three pairs of group primers listed in Table 3 were used for real-time RT-PCR. These primers correspond to three main categories of the *Trichoderma *cellulolytic enzyme systems that include cellobiohydrolase (CBH; exo-cellulases), endoglucanase (EGL; endo-cellulases) and beta-glucosidase (BGL). The gene expression profiling of the cellulases is shown in Figure 4B, with an increase between 6 to 18 weeks of composting, and a decrease thereafter. The expression patterns appeared to be similar among the tested genes during composting, an observation suggesting that these three types of cellulases may be expressed in a coordinated way that can enhance the overall efficiency of cellulose degradation.

Interestingly, as shown in Figure 4A-B, the gene expression of hemicellulases and cellulolytic system peaked at 15 and 18 weeks, respectively, suggesting that the microbial communities produce hemicellulases earlier than cellulases.

### (ii) Transcription-level profiling of fungal *LiPs *and *MnPs*

*Phanerochaete chrysosporium *is a model fungus that can degrade lignin without "touching" the cellulose of the wood. Like other white rot fungi, *P. chrysosporium *secretes an array of peroxidases and oxidases that attack lignin [30,34]. We have successfully designed primers, as listed in Table 3, for the genes encoding manganese peroxidases (MnPs) and lignin peroxidases (LiPs). Real-time RT-PCR was used in determining their expression levels.

**Figure 5:**
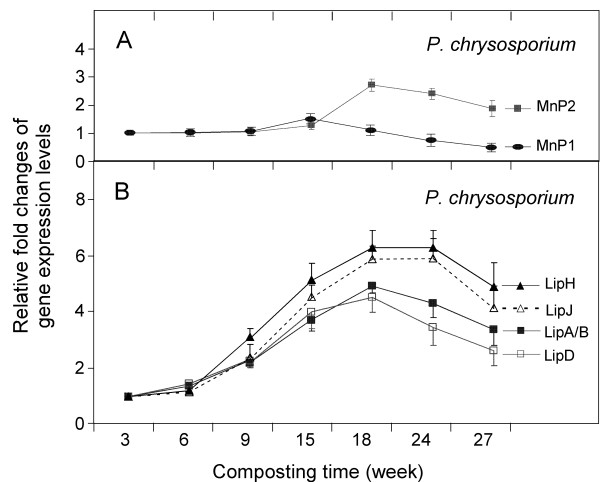
**Transcriptional level of representative lignin degradation-related genes during the composting of yellow poplar chips**. (**A**) Manganese peroxidase (*MnP*1 and *MnP*2). (**B**) Lignin peroxidase (*LiP *A/B, D, H and J). Gene sequences of fungus *Phanerochaete chrysosporium *were used to design primers for real-time RT-PCR. For each gene the expression level at each sampling time point of composting was compared to its expression level at 3 weeks (which was set as 1 fold).

As shown in Figure 5A, the maximal fold changes of *MnP*1 and *MnP*2 were relatively small, in that the peak of *MnP*1 expression was at 15 weeks with a 1.5-fold increase (relative to the expression level at 3 weeks), while *MnP*2 expression peaked later at 18 weeks with a larger (2.7-fold) increase. In contrast, the expression levels of the four *LiP *genes peaked at 18 weeks with more prominent changes than that of the *MnP *genes (Figure 5B). Peaks are observed in the expression levels for *LiP*A/B and *LiP*D at 18 weeks, whereas the fold-values for *LiP*H and *LiP*J, while also maximal at 18 weeks, maintained quite high expression levels at 24 weeks, indicating a longer high plateau for their expressions.

In this study, we examined the expression patterns of a total of six *P. chrysosporium *genes at seven sampling time points (from 3 weeks to 24 weeks yellow poplar composting); we glean from the expression profiling data that the two *MnP *genes are likely to be regulated differently, not only between themselves but also from the *LiPs *examined (Figure 5). This is in agreement with the findings by Janse et al. and Orth et al. who showed that *MnP*1-3 genes are genetically unlinked to each other or to any *LiP *genes [35,36].

### Hemicellulase and cellulase activities confirm microbial response to changes in chemical nature of exposed biomass surface

In addition to examining the expression levels of functional genes, another approach to studying the function of a microbial community is to measure the actual activities of enzymes that we are interested in (i.e., glycoside hydrolases, specifically cellulolytic and hemicellulolytic enzymes, among others). We used low-molecular-weight, soluble "model" substrates to assay activities in finely-ground samples of the total composted biomass materials, rather than in extracts. Our use of whole materials in the assays reflects our intention to conduct as comprehensive a survey as possible of the targeted glycoside hydrolase activities present in the composting material, including those activities tightly bound to the biomass as well as those readily extractable.

**Figure 6:**
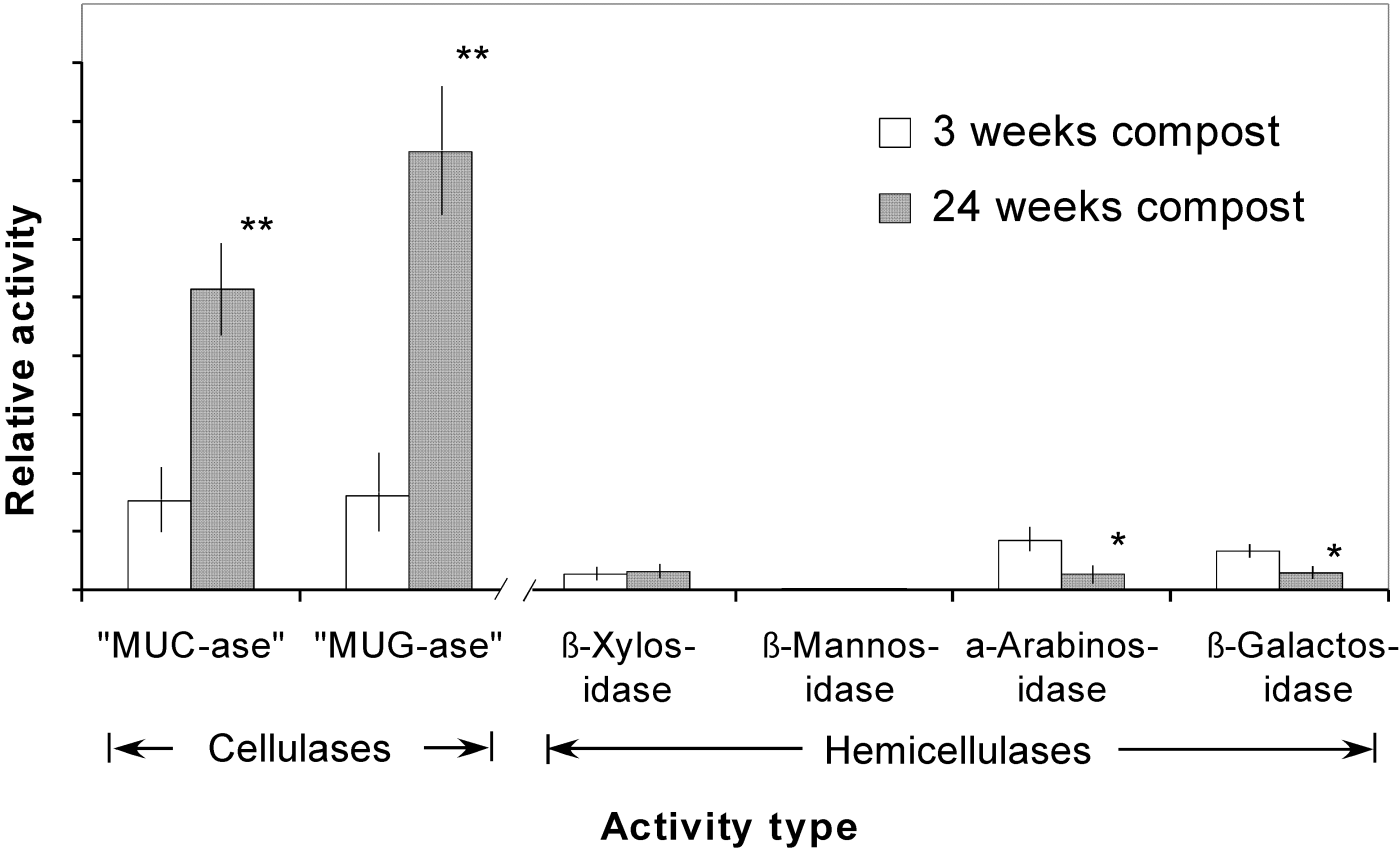
**Total cellulase and hemicellulase activities agaist model substrates measured in composted yellow poplar, as a function of composting time**. Activities are normalized to solids content of the compost sample and are averaged values from three replicates. Asterisks indicate statistically significant differences from the control (* for p < 0.05; ** for p < 0.01). Fluorogenic model substrates were used for the cellulase assay: MUC, 4-methylumbelliferyl-β-D-cellobioside; MUG, 4-methylumbelliferyl-β-D-glucoside. Hemicellulase assays utilized the respective 4-methylumbelliferyl-β-D-glycosides of the monosaccharides D-xylose, D-mannose, D-arabinose, and D-galactose.

Using fluorogenic model substrates, we found that the cellulase activities show increasing predominance in later stages (24 weeks) of composting (Figure 6). In contrast, the measured hemicellulase activities, mainly α-arabinosidase and β-galactosidase, were higher in the earlier stages (3 weeks). These results are consistent with the light and fluorescence microscope observations that showed celluloses are exposed mainly at the later stages of composting. These parallel optical and enzyme-activity surveys provide direct evidence that local microbial populations adjust their production of "harvesting" enzymes in response to the accessibility and digestibility of chemically different biomass materials (going after the more accessible and digestible materials first) and indirectly suggest that the makeup of the microbial population itself may change in response to the changes in the chemical and physical nature of the biomass as degradation proceeds.

## Discussion

### Potential impact of a mixed compost feedstock on the microbial community

Literature has shown that there is a correlation between microbial species composition and the types of substrates in biomass-degrading microbial community [37]. Although the mixing ratio in fresh weight is 1:1 for the two feedstocks (yellow poplar chips and mown lawn clippings) used in setting up our composters, their mixing ratio in dry weight is 6:1 (~85:15) after correction for the water content in each feedstock (listed in Table 4). Thus, yellow poplar is the major cellulolosic substrate based on its dry-weight contribution to the mixture.

**Table 4:** The fresh weight (FW) and dry weight (DW) mixing ratios, and the estimated recalcitrance index (RI) for yellow poplar chips and mown lawn clippings using in setting up the compost of this study

Compost feedstocks	FW mixing ratio	water %	DW mixing ratio	Estimated recalcitrance index *
Yellow Poplar	1	7%	6	0.56

Mown lawn	1	85%	1	0.25

Notes: *The RI value of yellow poplar chips is estimated to be 0.56, an average for hardwood biomass, while that of mown lawn clippings is estimated to be 0.25, a typical value for herbaceous plants such as corn stover and big bluestem grass biomass (see review [8]).

Furthermore, yellow poplar is much less degradable than mown lawn grass, with a recalcitrance index (RI) value twice that for the latter (Table 4). Taking these two considerations together, it is reasonable to conclude that the compost we characterized and presented here is dominated by yellow poplar substrate. In contrast, the impact of the minor feedstock, mown lawn grass, which accounts for only 15% of total dry feedstock in the setup of compost and is two times more easily degraded, is likely to be limited in determining the biochemical and microbial nature of the composting process, particularly in the later stages.

### Advantage and validity of using real-time PCR in analyzing transcriptional dynamics of composting

The challenge in characterizing the microbial population is choosing the suitable approach(es) to target specific microorganisms, microbial groups, or their functional genes. In literature, real-time PCR is a highly sensitive method that has been successfully used to quantify not only the bacterial amount in complex communities [25,38-41], but also the functional genes present in soil [42] and in lower termite gut [43]. Such versatility of the real-time PCR approach prompted us to apply this technique to estimate the abundance of microbial rDNA and the transcriptional levels of their functional genes in genomic DNA and mRNA samples from compost, respectively.

It is noteworthy that most of the RT-PCR primers developed in the current study were designed against the sequences of functional genes from a set of representative lignocellulolytic microbial species within a specific genus such as *Trichoderma *(Table 3 and Figure 4). Based on the fact that *Trichoderma *spp. are often the most prevalent culturable fungi in soils [44], it is reasonable to speculate that *Trichoderma *spp. are likely to be also relatively abundant in our compost. Nevertheless, future study is needed to quantify what portion of the composting community is accounted for by the *Trichoderma spp*., using genus-specific ITS rDNA primers. Equally important, further study is also needed to explore whether other prominent fungal taxa display similar or different functional gene expression profiles as the composting proceeds.

It should also be noted that despite our efforts to optimize the genomic DNA extraction procedure, it might still be possible that certain microbial species (like some archaea and fungi) may be underestimated because of differing ease of lysis of different microbial species. However, this should not affect the validity of the data analysis, as all the samples collected from different time points were subjected to the same DNA extraction method. However, other factors, such as the extensive cell lysis that may occur in the late composting phase and cause biased low DNA extraction yield, may contribute to the observed total genomic DNA decline in 24-27 weeks (Figure 3A).

### Correlative analysis in demystifying the composting process

The advantage of conducting a comprehensive investigation of composting is that it allows us to depict a multi-faceted picture of the structural, biochemical, and microbial dynamics involved in the natural degradation of yellow poplar wood chips. The combined use of a fluorescence labeling microscopic technique, cellulolytic enzyme assays, and real-time PCR allows us to make a correlative analysis. For example, the results showed that cellulase gene transcriptional levels peaked at 18 to 24 weeks of composting. In contrast, the transcriptional levels for surveyed hemicellulase genes peaked at around 15 weeks of composting. These results correlate well with the imaging data using GFP-tagged CBM as a molecular probe, which showed that the celluloses of yellow poplar biomass are mainly unwrapped and exposed at the later stage of composting (Figure 2), as well as with the enzymatic assay data, which showed the differential dominance of cellulase vs. hemicellulase in different stage of composting (Figure 6).

### Perspectives for biofuels production: lessons from analysis of microbial communities in compost

Consolidated bioprocessing (CBP) is a promising concept that integrates enzyme production, enzymatic hydrolysis, and fermentation into a single process step, in which a single microorganism with both ethanologenic and cellulolytic functions, or a microbial consortium that combines these functions, is used, usually in one reactor [1]. Several CBP microorganisms such as *Clostridium *spp., *Escherichia coli, Saccharomyces cerevisiae*, and *Bacillus subtilis *have been proposed and used to implement this concept [see review [45]]. We previously proposed the approach of using a microbial consortium rather than a single microorganism in the actual development of CBP technology and suggested a compatibility approach between cross-feeding and sugar transport in optimizing CBP based on general analysis of natural paradigms in plant cell wall deconstruction [8].

Composting in the current study can be viewed as a natural variant of CBP in rotary composters that involves the interactions of microbes and their secreted enzymes with plant cell walls. Here, we propose to incorporate into the strategy for the development of CBP one observed attribute of woody biomass compost, which is the observed fungal dominance in the late phase of composting when more cellulose is exposed. Such observation is in line with the natural degradation of biomass in wood litter with a higher fungal contribution (67-99%) to the total microbial mass (see review [8]). Because fungi have some types (families) of glycoside hydrolases (GHs), such as GH7 and GH61 [46,47], that bacteria lack, it is reasonable to speculate that the future CBP development may require transformation of fungi-unique GH genes and/or *P. chrysosporium*'s ligninase genes into CBP strains (so far mainly bacteria and yeasts) in order to further enhance their lignocellulolytic functions.

As the examined representative fungal xylanases, cellulases and ligninases were differentially expressed at different composting stages in this study, the timing of adding or expressing such enzymes in a CBP should also be more precisely regulated according to the progress of lignocellulosic substrate degradation.

Solid-state fermentation (SSF) is a process wherein the growth of microorganisms takes place on a solid substrate in absence or near absence of free water, but with enough moisture to support microbial growth and metabolism [48]. It has been playing an important role in the biomass conversion research, reflected by the fact that SSF has been used to produce cellulases using *T. reesei *[49-51] and *Penicillium janthinellum *[52], to produce xylanase using *B. subtilis *ASH [37,53], *B.licheniformis *A99 [54] and *Aspergillus tamarii *[55], and to produce multiple cellulolytic enzymes in *A. terreus *M11, using various lignocellulosic materials as carbon source [56]. Composting of woody biomass can be viewed as an SSF, and our observations suggest that by monitoring the gene as well as protein expression of cellulases and xylanases at different stages of SSF, one may optimize the harvest timing for different enzymes, depending on the enzyme targets. Recently, semi-solid state fermentation (with higher water contents than SSF) of the oleagenic fungus *Mortierella isabellina *on the substrates of crusted freshly harvested sweet sorghum have been used to produce biodiesel [57]. As *M. isabellina *cannot utilize cellulose and xylan, it is reasonable to speculate that a co-culture with lignocellulolytic fungi such as *T. reesei *and *P. chrysosporium *may reduce the substrate cost by increasing the portion of useable materials in the substrate mixture.

## Conclusion

To the best of our knowledge, this project constitutes the first comprehensive study of the natural composting of yellow poplar biomass. The following observations and implications highlight the value of using biomass compost for demystifying the enigma of "natural biomass conversion" in terms of lignocellulolytic gene expressions, enzymatic activities and their effects on deconstruction of plant biomass materials.

First, the *Ct*CBM3-GFP fluorescence labeling experiments, enzymatic activity analyses and functional gene expression profiling suggest that more hemicelluloses were degraded in the early stages of composting and that the celluloses in the biomass were thereby progressively more "unwrapped" and exposed at the later stages.

Second, a significant microbial population shift observed in this study suggests that studies of the conditions at the transition points between bacteria- and fungus-dominated stages are critical for identifying new microbial systems that are potentially applicable to biomass conversion. Future metatranscriptomic analysis via mass-scale sequencing during these stages and at their transitions can provide great potential for discovering novel cellulolytic microbes and enzymes.

## Materials and methods

### Apparatus and setup for biomass composting and sampling

A drum-shaped rotary composter (Figure 1A) was set up in October 2007. The composter was loaded with a 1:1 by fresh-weight mixture of yellow poplar sawdust chips (collected from Sawmiller Inc., Haydenville, Ohio) and freshly-mown lawn grass clippings. As listed in Table 4, the dry-weight mixing ratio of yellow poplar chips and mown lawn grass clippings is 6:1. The lawn grass used was Kentucky Bluegrass (*Poa pratensis *L.) and the lawn was not fertilized in 2007 or during the preceding year. To inoculate the sample with microbes, composted material taken from a mixed, already-established natural compost pile of trimmed tree branches and twigs was added. Water was added to moisten the sample, and after the entire contents of the composter were mixed thoroughly, the initial moisture content of the mixture was determined by oven-drying of representative aliquots, to be approximately 58%. The composter was rotated once a week, beginning on the 7^th ^day after the composter setup, in order to aerate the samples. At each of the designated sampling dates (i.e., 1, 3, 6, 9, 15, 18, 24, and 27 weeks), the composted material was thoroughly mixed by rotating the composter bin and by stirring the materials inside with bars, both horizontally and vertically. After mixing, a total of about 200 g of compost was sampled from various depths below the compost mass surface and mixed. An aliquot was air-dried and used to determine the water content, while the bulk of the withdrawn aliquot was stored at -80°C for later use in bio-imaging, microbial rDNA and gene transcriptional profiling (for the samples collected at all the sampling time points), and enzyme-activity analyses (for the samples collected at week 3 and 18) as well as chemical compositional analysis (for the samples collected at week 1 and 27). The presented data are based on the studies of the compost samples from October 2007 compost samples. To test the reproducibility of the major measurements of this study, a second independent composting experiment was conducted in August 2009 following the same procedure as described above. The same sampling patterns and analyses were carried out for the second composting to track its microbial community composition (rDNA profiling) and cellulolytic gene expression, and the results obtained were consistent with the observation we made by using the samples from October 2007 compost (data not shown).

### Measurement of temperature and oxygen concentration

Temperature and oxygen concentration were monitored constantly and recorded every other day during the composting process. Temperature at the center of the compost was measured using a 1522 Digital Indoor/Outdoor Thermometer (Taylor Precision Products, Oak Brook, IL) and a Windrow Thermometer (REOTEMP Instrument, San Diego, CA); the oxygen concentration was measured using an oxygen analyzer (Model 630; Engineered Systems & Designs, Newark, DE) at the center of the compost. To minimize the impact of the above-mentioned weekly (pre-sampling) rotation on the measurement of the temperature and oxygen concentration, these measurements were made before the scheduled rotation was conducted.

### Sample preparation for fluorescence microscopy

The structural changes in plant biomass produced by the microbial decay community was assessed by both white-light and fluorescence microscopy. For sample preparation, small amounts of the frozen composted samples were immersed in water to thaw and soften the material. Single pieces of the composted biomass were then chosen and hand-cut in an orientation that would result in a transverse cross-sectioning. The thin sections were kept in water until being deposited onto a glass coverslips for microscopic analysis. Chemically-specific labeling of the composted material utilized a family-3 carbohydrate-binding module (CBM) fused with a green-fluorescent-protein tag, *Ct*CBM3-GFP that was prepared in our previous work [20]. Labeling of the sectioned yellow poplar composted material with *Ct*CBM3-GFP was carried out in a blotting buffer (50 mM Tris, pH 8.0, 300 mM NaCl containing *Ct*CBM3-GFP at a concentration of 0.1-0.3 μg/μL) at room temperature for 30 min. The sample was washed three times in washing buffer (50 mM Tris, pH 8.0, 300 mM NaCl) followed by centrifugation (1000× g for 1 min). The final pellet was transferred to a slightly different buffer (50 mM Tris, pH 7.0, 20 mM NaCl) and subjected to microscopic analysis. Samples including original, untreated material as control, and material sampled at 6, 15, and 24 weeks composting, were imaged using an Olympus inverted fluorescence microscope. Representative images were selected for display based on analysis of 10-14 microscopic fields from each of the examined compost samples.

### Composted material compositional analysis

Compositional analysis of the composted materials was performed by using method described in the literature [58].

### Extraction of microbial genomic DNA and total fungal RNA from compost samples

Total microbial genomic DNA was extracted from 5-g compost samples using the standard procedure provided by the Ultra Clean Mega Soil DNA Kit (MO BIO Laboratories, Carlsbad, CA). To increase the purity of extracted genomic DNA, a further purification of DNA samples was conducted using the Qiagen DNA Purification Kit (QIAGEN USA, Valencia, CA). The prepared DNA samples were quantified using a Nanodrop 1000 Micro-Volume UV-vis Spectrophotometer (Thermo Fisher Scientific, Inc., Waltham, MA) and stored at -80°C until used for the real-time PCR analysis using microbial rDNA primers, as described later.

The total fungal RNA of the compost was extracted from 1 g of each compost sample, which was ground to fine powder in liquid nitrogen using a mortar and pestle, followed by the extraction and purification protocol for filamentous fungi using Qiagen RNeasy Plant Mini Kit (QIAGEN USA, Valencia, CA). The above extracted total fungal RNAs were treated with DNase I (Invitrogen, Carlsbad, CA) to eliminate the genomic DNA contamination. One microgram of purified total RNA was reverse-transcribed using SuperScript III Reverse Transcriptase with random primers (Invitrogen, Carlsbad, CA) according to the manufacturer's kit manual. The prepared fungal cDNA samples were stored at -20°C until used for the real-time RT (reverse transcription) PCR amplification using functional gene primers, as described later.

### Real-time PCR using total genomic DNAs and real-time RT PCR using fungal cDNA

Real-time PCR, using the universal primer sets for archaeal, bacterial and fungal rDNA (Table 2) and the abovementioned extracted genomic DNAs as templates, was used for the detection and relative quantification of archaea, bacteria, and fungi in the composted materials. It is noteworthy that for archaea and bacteria, the three rRNA genes (5 s, 16 s, and 23 s rDNA) typically exist as a co-transcribed operon. Similarly, fungi, like other eukaryotes, generally have many copies of the rRNA genes organized in tandem repeats; each repeat consists of the three genes encoding 5.8 s, 18 s, and 28 s rRNA, in which genes are present as one transcription unit separated by two internally transcribed spacers (ITS). The sequences of 16 s rDNA for archaea and bacteria and 5.8 s rDNA for fungi are highly conserved and thus are commonly used for phylogenic characterization of populations.

In parallel, real-time RT (reverse transcription) PCR was employed to profile the selected genes encoding cellulolytic enzymes over the time course of composting, using the above prepared fungal cDNAs as templates and the primers designed as follows. The designing of the primers (Table 3) for these genes were based on the available gene sequences of representative fungal genera or species. Except for the primers for ligninase-encoding genes, which are based on single species of *Phanerochaete chrysosporium*, all other primers for cellulase- and hemicellulase-encoding genes were based on sequences from 2-4 different species of the same genus, and can therefore be viewed as group primers at the sub-genus level (Table 3). These sequences were then used to design the primers using the program *Primer Express V.2 *(Applied Biosystems, Foster City, CA) and specifying a T_m _value between 58°C and 62°C and an amplicon size between 100 and 250 bp.

PCR analyses were performed using the iCycler iQ real-time detection system (Bio-Rad, Hercules, CA). The iCycler iQ optical system software (version 3.0a; Bio-Rad) was used to compile PCR protocols and set up the plate. Each microtiter plate well contained a 20-μL mixture of the following: 10 μL 1X iQ SYBR Green Supermix, which contained all the nucleotides, polymerase, reaction buffer, and SYBR green dye (Bio-Rad, Hercules, CA), 5 μL forward and reverse primers, giving final optimal concentrations of 300 nM for each, and 5 μL of DNA as template (2.5 ng per well). The PCR conditions for microbial rDNA amplification were described in the literature for the source of individual primers. For the primer pairs targeting functional genes listed in Table 3, amplification consisted of an initial hold at 95°C for 10 min followed by 40 cycles of 95°C for 15 s and 60°C for 60 s. All reactions were performed in triplicate and repeated in at least two independent experiments.

The PCR specificity for functional genes was confirmed at three levels. First, at the primer designing step, the designed primers were run through a Basic Local Alignment Search Tool (BLAST) search against the nucleotide collection (nr/nt) of NCBI. The designed primer should only hit only the target gene of the target species; any primer that matched sequences for organisms other than target species was abandoned. Second, at the end of real-time RT-PCR, the dissociation curve (i.e., melting curve) analysis of PCR end products was performed by ramping the temperature from 60°C to 95°C at a rate of 1°C per 30 s; a PCR reaction with high specificity should have a single melting peak. Third, PCR products were analyzed by agarose gel electrophoresis to check for the presence of single band, with some PCR product bands being gel-purified using QIAquick spin columns (QIAGEN USA, Valencia, CA) and sequenced.

### Enzyme assay of cellulase and hemicellulase activities

Approximately 5 g of frozen composted material was withdrawn from each of the representative samples at 3 weeks and 24 weeks into the composting process. Approximately 2.0 g of each sample was wet-ground with a mortar and pestle in 20 mM sodium acetate buffer, pH 5.0 at room temperature, then suspended in 20 mL of the same buffer to produce a uniform, readily-pipettable slurry. The remainder of each frozen compost sample was weighed, oven-dried (105°C, air), and reweighed to obtain a rough estimate of the biomass solids content of the slurry produced from that sample.

For the assay procedure, 0.8 mL of each well-mixed compost slurry was pipetted into each of three triplicate 2.0-mL HPLC vials. To each assay vial was added 0.2 mL of a 0.5-mM solution of a given 4-methyl-umbelliferyl-β-D-glycoside substrate (cellobioside, glucoside, arabinofuranoside, xylopyranoside, galactoside, or mannopyranoside from Sigma-Aldrich, St. Louis, MO) in 20 mM sodium acetate buffer, pH 5.0. Each vial was crimp-sealed, and the contents were mixed well by manual inversion before being incubated for 3 hours in a 30°C air incubator, with constant mixing by inversion at a rate of 10/min. At the end of the 3-h incubation, solids in the reaction mixtures were removed by centrifugation, and fluorescence of the cleavage product was developed by mixing 0.1 mL of each supernatant with 0.1 mL of 2 M Na_2_CO_3 _in one well of a 96-well, flat-bottomed polystyrene plate (Evergreen Scientific, Los Angeles, CA). Product fluorescence was then read in a Tecan GENios plate reader (Tecan US, Chapel Hill, NC) with excitation/emission wavelengths at 340/465 nm. Relative enzyme activities were reported as raw fluorescence readings, minus the fluorescence of an identically-treated blank with acetate buffer substituted for the biomass slurry.

## Abbreviations

BGL: Beta-glucosidase; CBH: Cellobiohydrolase; CBM: Carbohydrate-binding module; CBP: Consolidated bioprocessing; *Ct*CBM3: *Clostridium thermocellum *carbohydrate-binding module 3; EGL: Endoglucanase; ITS: Internal transcribed spacer; LiP: Lignin peroxidase; MnP: Manganese peroxidase; PCR: Polymerase chain reaction; rDNA or rRNA: ribosomal DNA or RNA; XYN: Xylanase.

## Competing interests

The authors declare that they have no competing interests.

## Authors' contributions

SYD, MPT and HW designed and coordinated the study, and revised the manuscript. HW, MPT and JOB conducted the compost setup and maintenance, sample harvesting, data analysis, and manuscript preparation. HW, MH and YL conducted DNA and RNA extraction, primer selection and PCR reactions. HW and QX carried out fluorescence microscopy study of composted samples. Enzymatic activity assay was carried out by JOB. MEH was consulted. All authors read and approved the final manuscript.
